# *Yellow-Green Leaf 19* Encoding a Specific and Conservative Protein for Photosynthetic Organisms Affects Tetrapyrrole Biosynthesis, Photosynthesis, and Reactive Oxygen Species Metabolism in Rice

**DOI:** 10.3390/ijms242316762

**Published:** 2023-11-25

**Authors:** Qiang Wang, Hongyu Zhang, Lingxia Wei, Rong Guo, Xuanzhi Liu, Miao Zhang, Jiangmin Fan, Siyi Liu, Jianglin Liao, Yingjin Huang, Zhaohai Wang

**Affiliations:** 1 Key Laboratory of Crop Physiology, Ecology and Genetic Breeding, Ministry of Education of the P.R. China, Jiangxi Agricultural University, Nanchang 330045, China; qiangwangchn@163.com (Q.W.); jn_zhanghongyu@163.com (H.Z.); wlx51204@163.com (L.W.); rong_guo1582@163.com (R.G.); jiangminfzz@163.com (J.F.); lsy64112138@163.com (S.L.); jlliao514815@163.com (J.L.); 2 Key Laboratory of Agriculture Responding to Climate Change, Jiangxi Agricultural University, Nanchang 330045, China; 3 College of Agronomy, Jiangxi Agricultural University, Nanchang 330045, China; liuxuanzhi000@163.com (X.L.); zhangmiao20231027@163.com (M.Z.)

**Keywords:** *yellow-green leaf 19* (*ygl19*), tetrapyrrole biosynthesis, photosynthesis, ROS metabolism, rice (*Oryza sativa*)

## Abstract

Chlorophyll is the main photosynthetic pigment and is crucial for plant photosynthesis. Leaf color mutants are widely used to identify genes involved in the synthesis or metabolism of chlorophyll. In this study, a spontaneous mutant, *yellow-green leaf 19* (*ygl19*), was isolated from rice (*Oryza sativa*). This *ygl19* mutant showed yellow-green leaves and decreased chlorophyll level and net photosynthetic rate. Brown necrotic spots appeared on the surface of *ygl19* leaves at the tillering stage. And the agronomic traits of the *ygl19* mutant, including the plant height, tiller number per plant, and total number of grains per plant, were significantly reduced. Map-based cloning revealed that the candidate *YGL19* gene was LOC_Os03g21370. Complementation of the *ygl19* mutant with the wild-type CDS of LOC_Os03g21370 led to the restoration of the mutant to the normal phenotype. Evolutionary analysis revealed that YGL19 protein and its homologues were unique for photoautotrophs, containing a conserved Ycf54 functional domain. A conserved amino acid substitution from proline to serine on the Ycf54 domain led to the ygl19 mutation. Sequence analysis of the *YGL19* gene in 4726 rice accessions found that the *YGL19* gene was conserved in natural rice variants with no resulting amino acid variation. The *YGL19* gene was mainly expressed in green tissues, especially in leaf organs. And the YGL19 protein was localized in the chloroplast for function. Gene expression analysis via qRT-PCR showed that the expression levels of tetrapyrrole synthesis-related genes and photosynthesis-related genes were regulated in the *ygl19* mutant. Reactive oxygen species (ROS) such as superoxide anions and hydrogen peroxide accumulated in spotted leaves of the *ygl19* mutant at the tillering stage, accompanied by the regulation of ROS scavenging enzyme-encoding genes and ROS-responsive defense signaling genes. This study demonstrates that a novel yellow-green leaf gene *YGL19* affects tetrapyrrole biosynthesis, photosynthesis, and ROS metabolism in rice.

## 1. Introduction

Rice (*Oryza sativa* L.) serves as a fundamental food crop, upon which a significant portion of the global population depends for sustenance, thereby rendering its yield crucial for ensuring worldwide food security [1]. The process of photosynthesis in plants serves as the primary means by which plants obtain energy, while chlorophyll and its derivatives play a pivotal role in photosynthesis, as chlorophyll captures light energy and facilitates its transfer to a reaction center [2]. A leaf color mutation can cause alterations in the content of photosynthetic pigments, thus impacting rice yield [3]. Leaf color mutants have been extensively studied in rice. The specific phenotypes of leaf color mutants are classified into eight types: *albino* (white), *xantha* (yellow), *viridis* (light green), *alboviridis* (upper portion white and lower portion green), *viridoalbina* (upper portion green and lower portion white), *xanthaviridis* (upper portion yellow and lower portion green), *viridoxantha* (upper portion green and lower portion yellow), and *striata* (chlorophyll deficiency in stripes) [4]. Based on the chlorophyll changes in the mutants, rice leaf color mutants are classified into four types: total chlorophyll-deficient, total chlorophyll increasing, chlorophyll *a*-deficient, and chlorophyll *b*-deficient [5].

The pigments of higher plants contain mainly chlorophyll *a* (Chl *a*), chlorophyll *b* (Chl *b*), and carotenoids [6,7]. The reaction center pigments both capture light energy and convert the absorbed light energy into electrical energy; the photosystem I (PSI) and photosystem II (PSII) peripheral light-trapping complexes contain most of the Chl *a* and all Chl *b* [8,9]. The light-trapping pigment absorbs and transmits light energy to the reaction center pigment [10]. Chlorophyll biosynthesis can be divided into two parts: tetrapyrrole biosynthesis and phytol chain biosynthesis [11,12]. The intermediate metabolites in the tetrapyrrole synthesis pathway are highly photoreactive sensitizers, and the accumulation of intermediate metabolites leads to the production of large amounts of reactive oxide species (ROS) [13], resulting in photodynamic damage and necrotic spots on developing leaves [14]. The tetrapyrrole metabolic pathway has been shown to be involved in plant spontaneous disease spot formation and, therefore, plant cells need to precisely regulate the tetrapyrrole metabolic pathway [15]. The tetrapyrrole biosynthetic pathway has been extensively studied in a variety of organisms through genetic and biochemical approaches, with each step of the reaction occurring in the plastid [16]. To date, the genes encoding the enzymes which catalyze various steps of the tetrapyrrole biosynthetic pathway have been successfully cloned in angiosperms, as represented by *Arabidopsis thaliana* [17,18,19]. The tetrapyrrole pathway for chlorophyll biosynthesis can be subdivided into two parts: biosynthesis from L-glutamyl-tRNA to protoporphyrin IX [20] and biosynthesis from protoporphyrin IX to chlorophyllide *a* and *b* [21,22].

Yellow-green leaf mutants can usually be used to clone the key enzyme genes in the chlorophyll synthesis pathway or important regulatory genes for chlorophyll metabolism. To date, more than 30 yellow-green leaf genes have been cloned in rice. *OsGluRS,* encoding the glutamyl-tRNA synthetase, is essential for glutamyl-tRNA synthesis in the chlorophyll synthesis pathway [23]. *OsChlI* and *OsChlD* separately encode the magnesium chelatase subunits ChlI and ChlD, participating in catalyzing the formation of Mg-protoporphyrin IX in the chlorophyll synthesis pathway [24]. *DVR* encodes the divinyl reductase that converts five divinyl substrates to corresponding monovinyl compounds in rice and is vital for chlorophyll biosynthesis [25]. *YGL1* encodes the chlorophyll synthase that catalyzes the conversion of chlorophyllide *a* to chlorophyll *a* in the chlorophyll synthesis pathway [26]. In addition to the key enzyme genes for chlorophyll synthesis mentioned above, more yellow-green leaf genes may be important regulatory genes for chlorophyll metabolism. *OsPAPST1* encodes a 3′-phosphoadenosine 5′-phosphosulfate carrier protein, which has been shown to act as a retrograde signal between chloroplasts and the nucleus [27]. *YGL8,* encoding a chloroplast-targeted UMP kinase, may affect the structure and function of chloroplast grana lamellae and indirectly influences chlorophyll synthesis [28]. *IspE*, encoding one of the seven known enzymes in the methylerythritol phosphate pathway, might affect chlorophyll metabolism through regulating the synthesis pathway of isoprenoids [29]. *LIL3,* encoding a light-harvesting like protein, is required for the stability of geranylgeranyl reductase, regulating chlorophyll metabolism by affecting phytyl biosynthesis [30]. *YL1,* encoding a plant lineage-specific auxiliary factor involved in the biosynthesis of the chloroplast ATP synthase complex, affects chlorophyll accumulation by regulating photosynthesis [31]. These observations exemplify how leaf color-related genes are only a small fraction of the cloned genes regulating chlorophyll metabolism. And all identified leaf color-related genes might also be insufficient to adequately elucidate the regulatory mechanism of chlorophyll metabolism, which needs more genes to be cloned.

In this study, a spontaneous mutant, *yellow-green leaf 19* (*ygl19*), was isolated from rice. And morphological identification, genetic analysis, gene cloning, and function analysis were carried out. Based on phenotypic and molecular characterizations, the *YGL19* gene appears to play multiple important roles in tetrapyrrole biosynthesis, photosynthesis, and ROS metabolism.

## 2. Results

### 2.1. Phenotypic Characterization of the ygl19 Mutant

**Figure 1:**
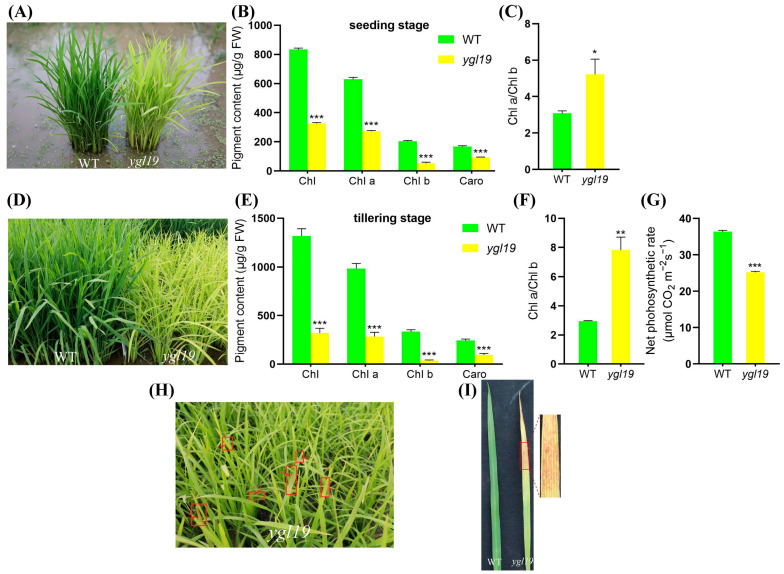
Phenotypic comparison of wild-type and *ygl19* mutant plants. (**A**) Plants at the seedling stage in the paddy field. (**B**) Contents of photosynthetic pigments (Chl, Chl *a*, Chl *b,* and Caro) in leaves at the seedling stage. (**C**) Chlorophyll *a*/*b* ratio at the seedling stage. (**D**) Plants at the tillering stage in the paddy field. (**E**) Contents of photosynthetic pigments (Chl, Chl *a*, Chl *b,* and Caro) in leaves at the tillering stage. (**F**) Chlorophyll *a*/*b* ratio at the tillering stage. (**G**) Comparison results of net photosynthetic rate. (**H**) Phenotype of the *ygl19* plants and lesion leaves at the tillering stage (indicated by red arrow). (**I**) Spot phenotype on *ygl19* leaves with wild type as control. WT, wild type. All data represent the mean ± SD of three biological replicates, and asterisks indicate statistically significant differences between *ygl19* and wild-type plants (* *p* < 0.05, ** *p* < 0.005, *** *p* < 0.0005, Student’s *t*-test).

The spontaneous mutant *ygl19* was isolated from the wild-type indica rice cultivar Shuangyinzhan and exhibited the yellow-green leaf phenotype at the seedling stage (Figure 1A). Meanwhile, *ygl19* had a more significant leaf color difference at the tillering stage (Figure 1D). In order to further clarify the yellow-green leaf phenotype of the *ygl19* mutant, the photosynthetic pigment contents of the mutant and its wild type were determined at different stages. At the seedling stage, the contents of total chlorophyll (Chl), Chl *a*, Chl *b*, and carotenoids (Caro) in the leaves of the *ygl19* mutant were significantly reduced by 60.9%, 56.7%, 74.0%, and 44.3%, respectively, compared with those in the wild-type leaves, with Chl *a*/Chl *b* increased by 41.0% (Figure 1B,C). At the tillering stage, the total Chl, Chl *a*, Chl *b*, and Caro contents in the mutant leaves were more significantly decreased by 75.6%, 71.1%, 89.1%, and 60.4%, respectively, with Chl *a*/Chl *b* increased by 62.5% (Figure 1E,F). And comparing the results of net photosynthetic rate showed that the net photosynthetic rate of the *ygl19* mutant was significantly decreased compared to the wild type (Figure 1G). These results suggested that the yellow-green leaf phenotype of the *ygl19* mutant was due to a defect in the photosynthetic pigments, which might further affect the photosynthetic performance. Interestingly, at the tillering stage, reddish-brown spots appeared on the *ygl19* mutant leaves (Figure 1H,I); however, this phenomenon would no longer appear when the *ygl19* mutant developed into the heading stage.

**Figure 2:**
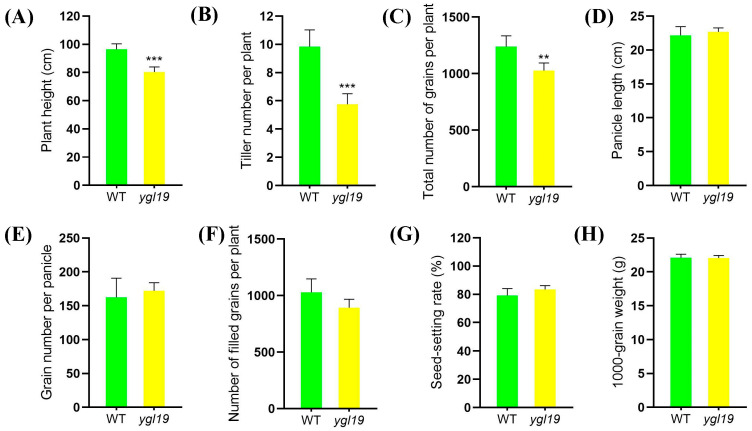
Comparison of agronomic characters of wild-type and *ygl19* mutant plants. (**A**) Panicle length. (**B**) Tiller number. (**C**) Total number of grains per plant. (**D**) Panicle length. (**E**) Grain number per panicle. (**F**) Number of filled grains per plant. (**G**) Seed setting rate. (**H**) 1000-grain weight. WT, wild type. All data represent the mean ± SD of three biological replicates, and asterisks indicate statistically significant differences between *ygl19* mutant and wild type (** *p* < 0.005, *** *p* < 0.0005, Student’s *t*-test).

Eventually, agronomic traits of the *ygl19* mutant and wild type at the maturation stage were investigated. The results showed that the differences in the plant height, tiller number per plant, and total number of grains per plant between the wild type and the *ygl19* mutant were obvious, and those of the *ygl19* mutant were significantly reduced by 16.7%, 17.1%, and 41.5%, respectively, compared with those of the wild type (Figure 2A–C). However, the panicle length, grain number per panicle, number of filled grains, seed setting rate, and 1000-grain weight showed no difference between the *ygl19* mutant and wild type (Figure 2D–H).

### 2.2. Map-Based Cloning of the YGL19 Gene

For the genetic analysis, two hybrid populations were separately constructed from the crosses between the *ygl19* mutant with 02428 and 9311. All F_1_ plants from these crosses displayed wild-type green leaves, while F_2_ progenies of both the *ygl19*/9311 and *ygl19*/02428 populations showed a segregation ratio of 3:1 (χ^2^_3:1_ < χ^2^_0.05_ = 3.84, *p* > 0.05, Table 1) for wild-type green leaves to yellow-green and spotted leaves, which confirmed that a single recessive nuclear gene is responsible for the yellow-green leaf and leaf spot phenotype of the *ygl19* mutant.

**Figure 3:**
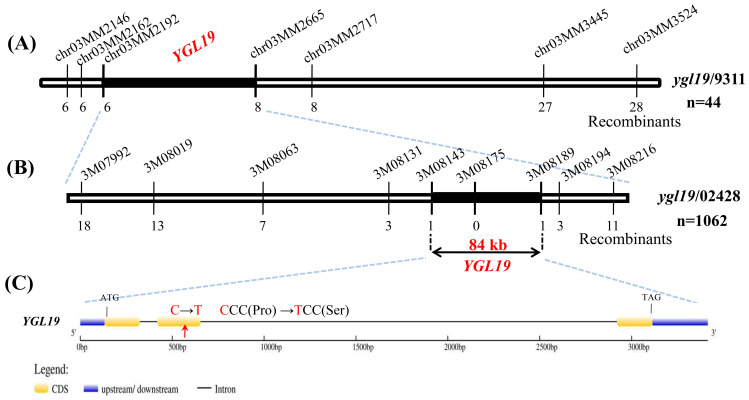
Map-based cloning of the *YGL19* gene. (**A**) Preliminary mapping of the *YGL19* gene. The *YGL19* locus was initially mapped to a 3.3 Mb region between two markers, chr03MM2192 and chr03MM2665, on chromosome 3. (**B**) Fine mapping of the *YGL19* gene. The locus was further mapped within an 84 kb region between the 3M08143 and 3M08189 markers. Numbers below the line indicate the number of F_2_ recombinants at the marker regions. (**C**) The C to T substitution of the *YGL19* gene in *ygl19* mutant. Yellow and blue squares represent the CDS and upstream/downstream region, respectively. The black line represents the intron. The red arrow indicates the C to T substitution position in *ygl19* mutant. The codon changed from CCC to TCC, and the amino acid coding changed from Proline (Pro) to Serine (Ser).

The F_2_ population produced by crossing the mutant *ygl19* with 9311 was used for the preliminary mapping of the *YGL19* gene. Using 44 F_2_ mutant plants with yellow-green leaf and leaf spots, the *YGL19* locus was mapped to a 3.3 Mb region between the SSR makers chr03MM2192 and chr03MM2665 on chromosome 3 (Figure 3A), and it was hard to find a polymorphism marker between the *ygl19* mutant and 9311 in this region. However, nine polymorphism markers (3M07992, 3M08019, 3M08063, 3M08131, 3M08143, 3M08175, 3M08189, 3M08194, and 3M08216) were developed in this region between the *ygl19* mutant and 02428. Accordingly, using 1062 F_2_ mutant plants with yellow-green leaf and leaf spots generated from the cross of the *ygl19* mutant and 02428, the *YGL19* locus was fine-mapped to an 84 kb region flanked by markers 3M08143 and 3M08189 (Figure 3B). Twenty-seven putative genes were predicted using the Rice Annotation Project Database (https://rapdb.dna.affrc.go.jp/, accessed on 23 December 2019) in the candidate region. By sequencing these genes of the *ygl19* mutant, a 340th bp C→T substitution was found in the second exon of the gene LOC_Os03g21370, resulting in an amino acid alternation of proline (Pro) to serine (Ser) (Figure 3C). As a result, LOC_Os03g21370 is the likely candidate of the *YGL19* gene.

### 2.3. A Complementation Experiment of LOC_Os03g21370 into the ygl19 Mutant

**Figure 4:**
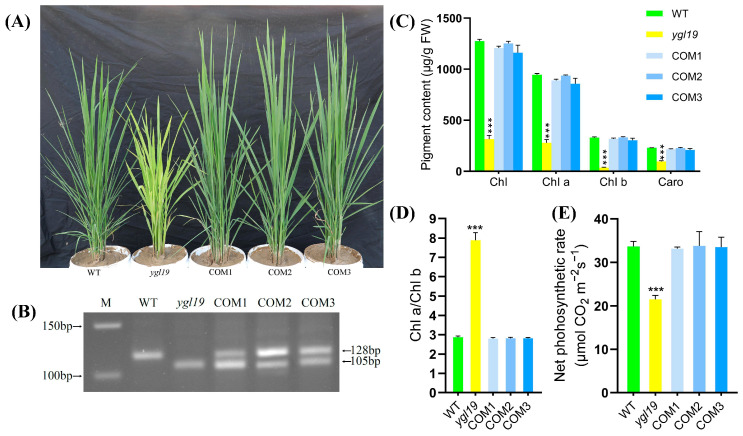
Complementation of the *YGL19* gene into the *ygl19* mutant. (**A**) From left to right: plants of wild type, the *ygl19* mutant, and three independent *YGL19* T_0_ complementation transgenic lines at the tillering stage. (**B**) The dCAPs molecular marker identification of the *YGL19* gene and the *ygl19* gene in plants corresponding to (**A**). (**C**) Contents of photosynthetic pigments (Chl, Chl *a*, Chl *b,* and Caro) in leaves of wild type, the *ygl19* mutant, and *YGL19* T_1_ generation of complementation transgenic lines at the tillering stage. (**D**) Chlorophyll *a*/*b* ratio of T_1_ generation of complementary transgenic lines at the tillering stage. (**E**) Net photosynthetic rate of T_1_ generation of complementary transgenic lines at the tillering stage. WT, wild type. COM1, COM2, and COM3: three representative *YGL19* complementary transgenic lines. All data represent the mean ± SD of three biological replicates, and asterisks indicate statistically significant differences between the *ygl19* and wild-type plants (*** *p* < 0.0005, Student’s *t*-test).

In order to further confirm that the yellow-green leaf phenotype of the *ygl19* mutant was caused by the mutation in the *YGL19* gene, complementary vectors with the *YGL19* gene were constructed to carry out a complementary transgenic experiment. In detail, the full-length coding sequence of the wild-type *YGL19* gene (LOC_Os03g21370) was amplified and inserted into the pBWA(V)HU vector following the Ubi promoter. Then, the fusion vector pBWA(V)HU-Ubi-YGL19 was introduced into the *ygl19* mutant calli via *Agrobacterium tumefaciens*-mediated transformation. As a result, a total of 10 positive transgenic lines were obtained through identification with a PCR amplification of the screening resistance gene (Figure S1). These complementary transgenic lines all showed the normal green leaf color phenotype as the wild type (Figure 4A), with no leaf spot appearing during the whole tillering stage. And the success of the complementary transgene was identified via dCAPs determination with both wild-type and *ygl19* mutant bands in transgenic lines (Figure 4B). Analysis of the pigment contents and photosynthetic performance showed that total Chl, Chl *a*, Chl *b*, Caro, Chl *a*/Chl *b* ratio, and net photosynthetic rate of the complementary transgenic lines were all restored to normal, as did those in the wild-type plants (Figure 4C–E).

**Figure 5:**
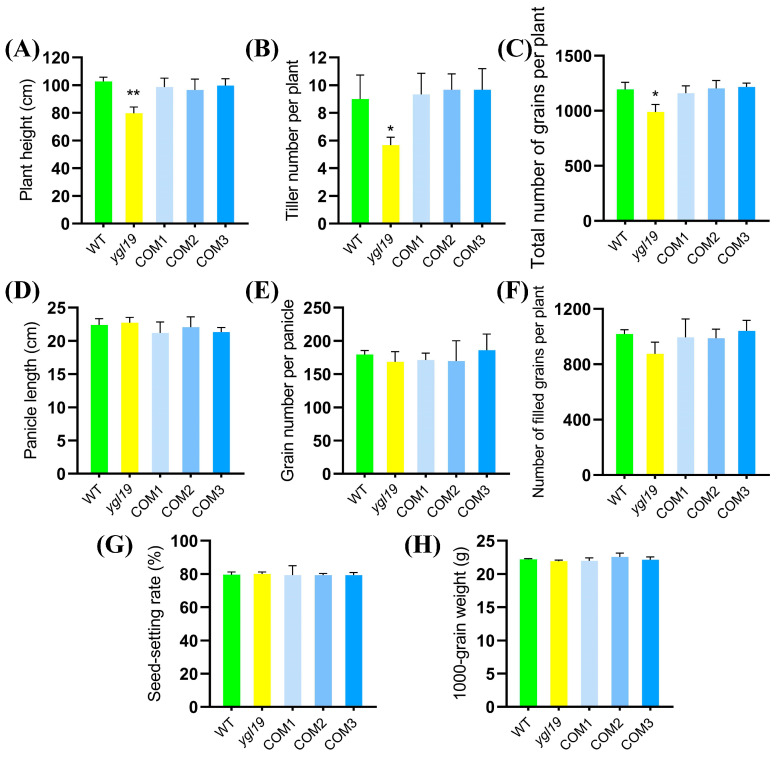
Agronomic characters of the *YGL19* gene complementation. (**A**) Plant height. (**B**) Tiller number. (**C**) Total number of grains per plant. (**D**) Panicle length. (**E**) Grain number per panicle. (**F**) Number of filled grains per plant. (**G**) Seed setting rate. (**H**) 1000-grain weight. WT, wild type. COM1, COM2, and COM3: three representative *YGL19* complementary transgenic lines at T1 generation. All data represent the mean ± SD of three biological replicates, and asterisks indicate statistically significant differences between the *ygl19* and wild-type plants (* *p* < 0.05, ** *p* < 0.005, Student’s *t*-test).

Additionally, the agronomic traits were investigated. The results showed that the differences for plant height, tiller number per plant, and total number of grains per plant between the *ygl19* mutant and wild type were also all restored to normal in complementary transgenic lines (Figure 5A–C). In summary, functional complementation of the wild-type *YGL19* gene rescued the mutant phenotype of the *ygl19* mutant, confirming the fact that LOC_Os03g21370 is indeed the *YGL19* gene.

### 2.4. The YGL19 Protein Homologs Are Unique for Photosynthetic Organisms and Are Evolutionarily Conserved, Containing a Ycf54 Functional Domain

**Figure 6:**
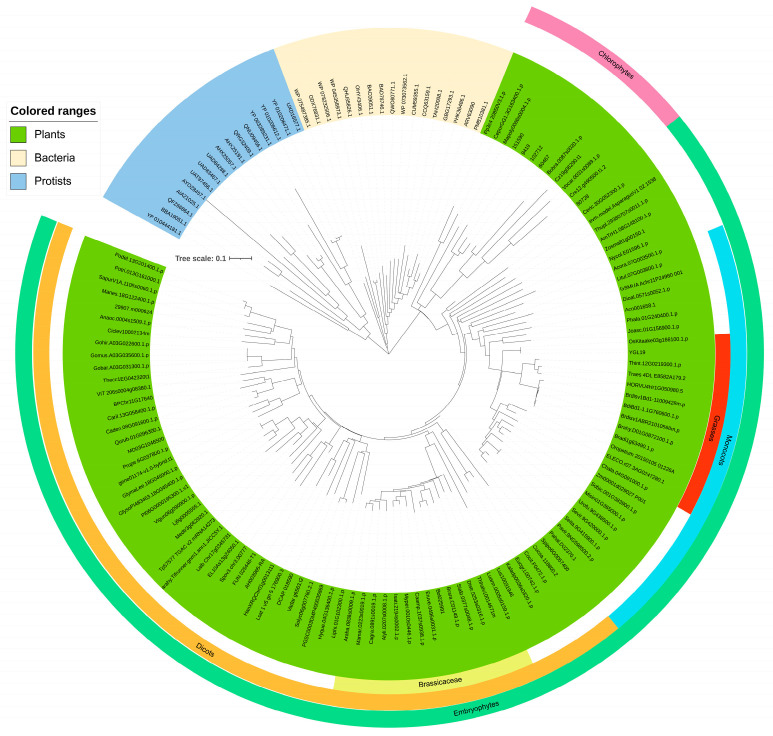
Phylogenic tree of the YGL19 protein and its homologs. Protein sequences of 139 species (including 106 green plants, 16 protists, and 17 bacteria) were used to construct phylogenetic trees. The tree was constructed using MEGA version 7.0. Statistical support for the nodes is indicated and the scale bar represents percentage substitutions per site. The scale of phylogenetic tree is 0.05. Further legend information is provided in Supplementary Table S3.

The plant homologs of the YGL19 protein were blasted in phytozome (https://phytozome-next.jgi.doe.gov/, accessed on 16 November 2022), and other homologs were retrieved in NCBI (https://blast.ncbi.nlm.nih.gov/Blast.cgi, accessed on 16 November 2022). These YGL19 homologs are ubiquitous in all representative species of plants, while animals and protozoa do not appear to contain these homologs (Supplementary Table S3). The YGL19 homologs in protists were clustered into brown algae and red algae, and homologs in bacteria were in both genera of cyanobacteria, such as *Microcystis aeruginosa*, *Oscillatoriales cyanobacterium*, *Prochlorococcus marinus,* and *Synechocystis* sp. PCC 6803. Brown algae, red algae, and cyanobacteria could obtain energy via photosynthesis. Therefore, YGL19 protein homologs are unique for photosynthetic organisms. To study the origin and evolutionary history of YGL19 protein homologs in different species, a phylogenetic tree was constructed with the neighbor-joining method (Figure 6). These YGL19 homologs were clustered into three large groups, containing plants, protists, and bacteria. YGL19 homologs in plants were tightly clustered into two separate groups, embryophytes and chlorophytes, with the former sub-clustered into monocots and dicots, indicating that gene divergence occurred after the diversification of these clades. Then, the dicots contained 14 brassicaceae and 13 monocot species, which were classified into grasses containing the YGL19 protein. Among all orthologous proteins, the YGL19 protein exhibited the most significant similarity to the homolog of *Oryza sativa Kitaake* (*OsKitaake03g166100.1.p*). These results suggested that the YGL19 protein homologs were evolved from lower light-autotrophic organisms and existed only in photosynthetic organisms, with them not being present in nonphotosynthetic organisms, possibly playing a crucial role in the energy metabolism process of Earth’s organisms.

**Figure 7:**
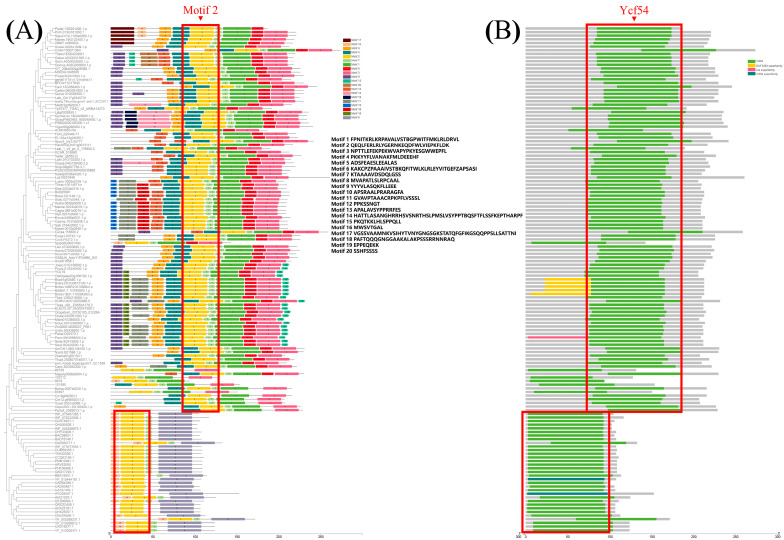
Comparison of motifs and Ycf54 domain of 139 YGL19 homologs from 139 species. (**A**) Motif 2 exists in the Ycf54 domain of 139 species and is labeled in a rectangular box. (**B**) Ycf54 domain exists in 139 species and is labeled in a rectangular box. The right rectangles with different colors show motif and superfamily names. Black text denotes motif amino acid sequences.

The motifs of YGL19 homologs from 139 species were further analyzed based on the primary protein sequences using MEME software v5.3.0 (Figure 7A). Motif 2 existed in all species. Motifs 7, 4, 1, 5, and 3 were unique sequences for all or most plants, while motifs 10 and 6 were unique sequences at the N-terminus and C-terminus in protists and bacteria. Moreover, motifs 15 or 16 were unique sequences in 14 brassicaceae, while motif 13 was the special sequence for most monocot species, including rice with the YGL19 protein. All the above contributed to the conserved functional evolutionary relationships of YGL19 homologs and diverged them into two large clades (plant and others). Meanwhile, four domains were predicted in these species, including Ycf54, the DUF3363 superfamily, the rne superfamily, and the Ycf54 superfamily (Figure 7B). The Ycf54 domain is displayed in all species except three bacteria, which have the Ycf54 superfamily instead of Ycf54. Moreover, the DUF3363 superfamily was only predicted at the N-terminus of Brachypodium, which might lead to the branching evolution. Notably, the rne superfamily only belongs to one branch of Panicoideae, named *Panicum virgatum* (*Pavir.9NG598500.2.p*). It is speculated that the conserved motifs and domains of YGL19 homologs in different species play the essential roles for photosynthetic organisms, while the additional motifs and domains are also indispensable, possibly playing a key role in their functional specificity.

### 2.5. A Conserved Amino Acid on the Ycf54 Domain Is Substituted to Form the ygl19 Protein

**Figure 8:**
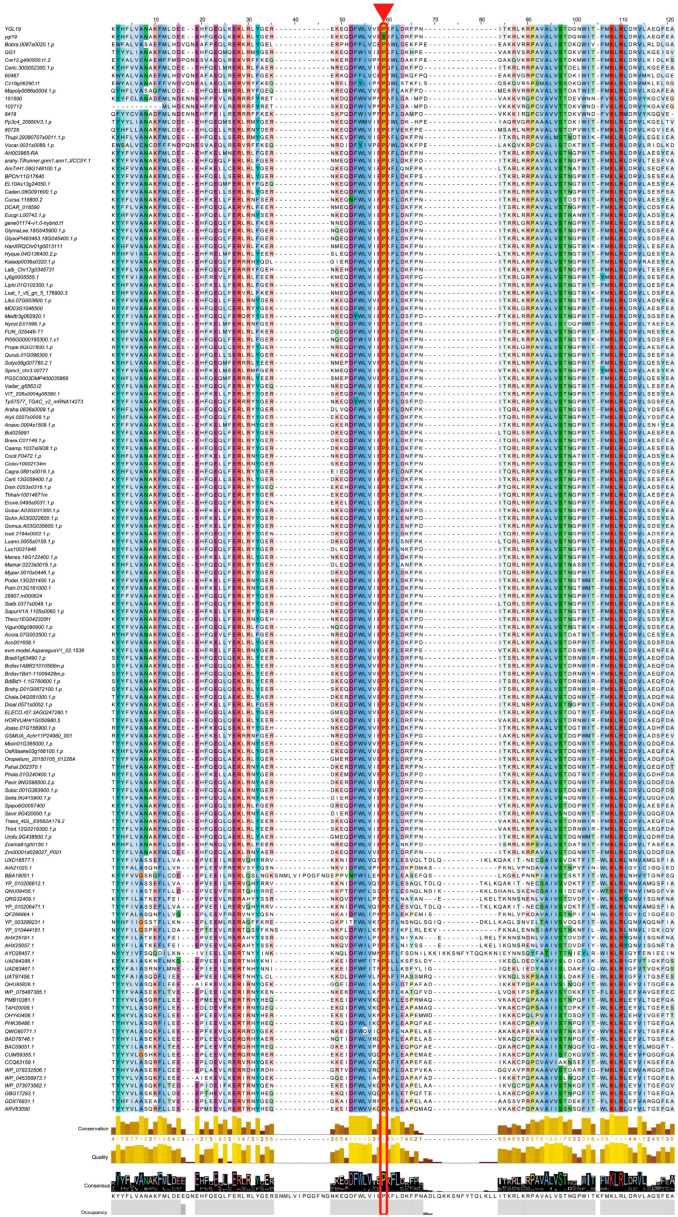
Multiple sequence alignment of the YGL19 protein and ygl19 protein with their homologs in different photosynthetic organisms (total of 140 protein sequences). The amino acid substitution of Pro (P) to Ser (S) at position 59 in ygl19 protein sequences is labeled with a red triangle and in a rectangular box. Color background indicates positions conserved in >30% of the aligned sequences; color depth indicates the degree of conservation.

A multiple sequence alignment analysis was conducted for the Ycf54 domain of YGL19 homologs in 139 species, including the wild-type rice with the YGL19 protein and the *ygl19* mutant rice with the ygl19 protein (a total of 140 protein sequences). The result showed most amino acids were highly conserved among all analyzed species, and two amino acids, proline (P at the position 59) and lysine (K at the position 108), were originally fully conserved among all species (Figure 8), implying their biologically important roles for YGL19 homologs. Interestingly, the proline (P) to serine (S) substitution from the YGL19 protein to ygl19 protein is exactly the proline at position 59 (Figure 8).

### 2.6. The YGL19 Gene Is Conserved in a Natural Rice Variant

To address the question of whether the amino acid sequences are conserved for the *YGL19* gene, we scanned the gene sequences of *YGL19* in 4726 rice accessions in RiceVarMap. The results showed that there were only 3 mutations in the exons of the *YGL19* gene, all of which were synonymous mutations without amino acid variation; 20 mutations were found in introns, 1 at 5′ UTR, and 1 at 3′ UTR, with none leading to amino acid variation (Table 2). For these three synonymous variants, they all showed relatively high prevalence in nature. Among them, the 16th amino acid was Ala with a primary allele frequency of 60.4%. The 63rd amino acid was Asp with a primary allele frequency of 60.3%. And the 111th amino acid was Val with a primary allele frequency of 59.9%. The results implied that the *YGL19* gene is extremely conserved in rice varieties, and its amino acid mutation was not reserved under natural evolution and artificial domestication. The *YGL19* gene might be extremely difficult to mutate in rice, or its mutations might not be popular with rice breeders, possibly attributing to their abnormal growth and development.

Meanwhile, we investigated the 2 kb promoter region of the *YGL19* gene, and 57 variations were found from the promoter region (Supplementary Table S4). In these upstream variations, we found 9 indel variations and 48 SNP variations. The variations in the promoter regions had different types, which might exert the *YGL19* gene’s function via transcription regulation.

### 2.7. The YGL19 Gene Is Mainly Expressed in Green Tissues and Localized in Chloroplasts for Function

**Figure 9:**
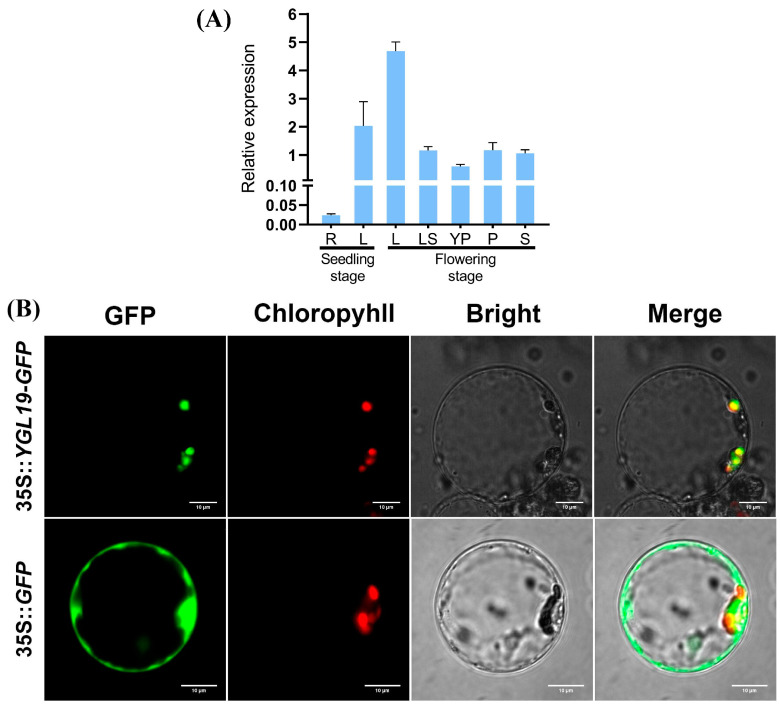
Tissue expression pattern of the *YGL19* gene and subcellular localization of the YGL19 protein in rice. (**A**) Relative expression level of the *YGL19* gene in different tissues, including root (R) and leaf (L) at the seedling stage, and leaf (L), leaf sheath (LS), unflowered young panicle (YP), flowering panicle (P), and stem (S) at the flowering stage. Data represent the mean ± SD of three biological replicates. (**B**) Rice protoplast transformed with 35S::*YGL19*-*GFP*, and the empty vector 35S::*GFP* as control. Green fluorescence shows GFP; red fluorescence indicates chlorophyll autofluorescence in chloroplast; yellow fluorescence indicates images with the two types of fluorescence merged; and bright field images show rice protoplasts (Bars = 10 μm).

To explore the expression pattern of the *YGL19* gene, its transcription level was examined via qRT-PCR in different wild-type tissues (Figure 9A). The results showed that the *YGL19* gene is expressed in a wide range of tissues, including the roots and leaves at the seedling stage, and the stem, leaves, leaf sheaths, unflowered young panicles, and flowering panicles at the flowering stage. Nonetheless, the *YGL19* gene is differentially expressed in different tissues. Specifically, leaves showed the highest expression of the *YGL19* gene, with its relative expression in the flag leaf at the flowering stage being higher than that at the seedling stage. Subsequently, the leaf sheath, flowering panicle, stem, and young panicle showed moderate expression of the *YGL19* gene. Meanwhile, roots showed very few expressions of the *YGL19* gene. These results suggest that the *YGL19* gene is mainly expressed in green tissues, especially in leaf organs, for function.

To reveal the subcellular localization where the YGL19 protein functions in the cell, a 35S::*YGL19*-*GFP* fusion expression vector was constructed. Then, the 35S::*YGL19*-*GFP* vector and the empty vector control 35S::*GFP* were separately introduced into rice protoplasts to conduct a transient expression assay and were observed via a laser scanning confocal microscopy. The results showed that the green fluorescence of the YGL19-GFP fusion protein completely overlapped with the red autofluorescence of chlorophyll in the chloroplast. At the same time, the green fluorescence of the empty vector was diffusely distributed in the cytoplasm as expected (Figure 9B). These results confirmed that the YGL19 protein targets the chloroplast to perform its function.

### 2.8. Expression Analysis of the Genes for Tetrapyrrole Biosynthesis in the ygl19 Mutant

**Figure 10:**
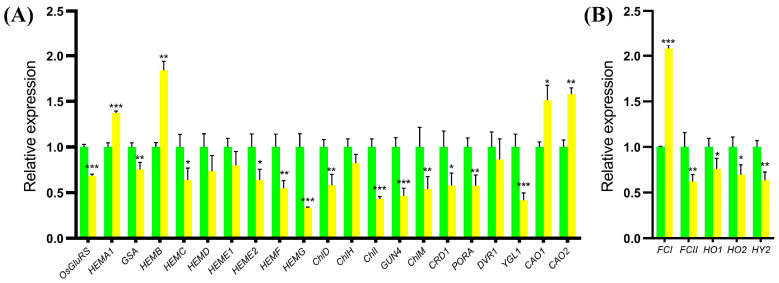
Relative expression of tetrapyrrole synthesis-related genes in wild-type and *ygl19* plants. (**A**) Genes in chlorophyll synthesis branch. (**B**) Genes in heme synthesis branch. The expression level of each gene in the wild type was set to 1.0, and those in the *ygl19* mutant were calculated accordingly. Data represent the mean ± SD of three biological replicates, and asterisks indicate statistically significant differences between the *ygl19* mutant and wild-type plants (* *p* < 0.05, ** *p* < 0.005, *** *p* < 0.0005, Student’s *t*-test).

Since the chlorophyll contents of the *ygl19* mutant reduced, the expression changes in tetrapyrrole biosynthesis-related genes in wild-type and *ygl19* mutant seedling leaves were detected via qRT PCR. The tetrapyrrole biosynthesis pathway contains two branches, the chlorophyll synthesis branch and the heme synthesis branch. For the detected genes in the chlorophyll synthesis branch, four genes (*HEMA1*, *HEMB*, *CAO1,* and *CAO2*) were upregulated in the *ygl19* mutant; meanwhile, 13 genes (*OsGluRS*, *GSA*, *HEMC*, *HEME2*, *HEMF*, *HEMG*, *ChlD*, *ChlI*, *GUN4*, *ChlM*, *Mpe*, *POR1,* and *YGL1*) showed downregulation in the *ygl19* mutant (Figure 10A). For the detected genes in the heme synthesis branch, one gene, *FCI,* was up regulated in the *ygl19* mutant; meanwhile, four genes (*FCII*, *HO1*, *HO2,* and *HY2*) showed downregulation in the *ygl19* mutant (Figure 10B). This molecular evidence suggests that tetrapyrrole biosynthesis-related genes were regulated to affect the contents of photosynthetic pigments in the *ygl19* mutant.

### 2.9. Expression Analysis of the Genes for Photosynthesis in the ygl19 Mutant

**Figure 11:**
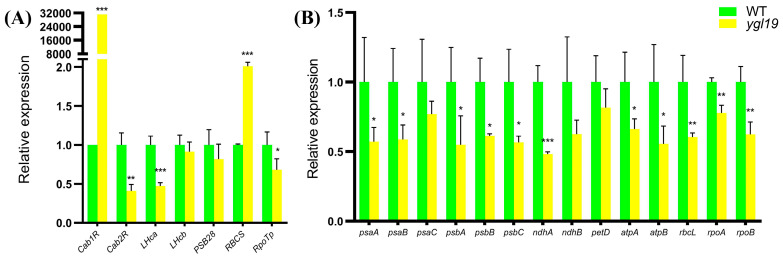
Relative expression of photosynthesis-related genes in wild-type and *ygl19* mutant plants. (**A**) Nucleus-encoded photosynthesis-related genes. (**B**) Plastid-encoded photosynthesis-related genes. The expression level of each gene in the wild type was set to 1.0, and those in the *ygl19* mutant were calculated accordingly. WT, wild type. Data represent the mean ± SD of three biological replicates, and asterisks indicate statistically significant differences between the *ygl19* mutant and wild-type plants (* *p* < 0.05, ** *p* < 0.005, *** *p* < 0.0005, Student’s *t*-test).

The expression levels of nucleus- and plastid-encoded photosynthesis-related genes were detected in wild-type and *ygl19* seedling leaves via qRT-PCR. The detected nucleus-encoded photosynthesis-related genes contained genes encoding chlorophyll *a*/*b* binding protein (*Cab1R* and *Cab2R*), light-harvesting Chl *a*/*b*-binding protein of PSII (*LHca* and *LHcb*), PSII reaction center protein (*PSB28*), Rubisco small subunit (*RBCS*), and plastid RNA polymerase (*RpoTp*). The results showed that *Cab1R* and *RBCS* were upregulated in the *ygl19* mutant, while *Cab2R*, *LHca* and *RpoTp* were downregulated in the *ygl19* mutant (Figure 11A). The detected plastid-encoded photosynthesis-related genes contained genes encoding the subunit of photosystem I (*psaA*, *psaB* and *psaC*), core component of photosystem II (*psbA*, *psbB* and *psbC*), NADH dehydrogenase subunit (*ndhA* and *ndhB*), cytochrome *b_6_f* complex subunit (*petD*), ATP synthase complex subunit (*atpA* and *atpB*), Rubisco large subunit (*rbcL*), and plastid RNA polymerase subunit (*rpoA* and *rpoB*). The results showed that *psaA*, *psaB*, *psaC*, *ndhA*, *ndhB*, *petD*, *atpA*, *atpB*, *rbcL*, *rpoA,* and *rpoB* were all downregulated in the *ygl19* mutant. This molecular evidence implies that nucleus- and plastid-encoded photosynthesis-related genes were regulated to mediate photosynthetic performance in the *ygl19* mutant.

### 2.10. ROS Accumulation, the Regulation of ROS-Scavenging Genes, and ROS-Responsive Defense Signaling Genes in ygl19 Leaves

**Figure 12:**
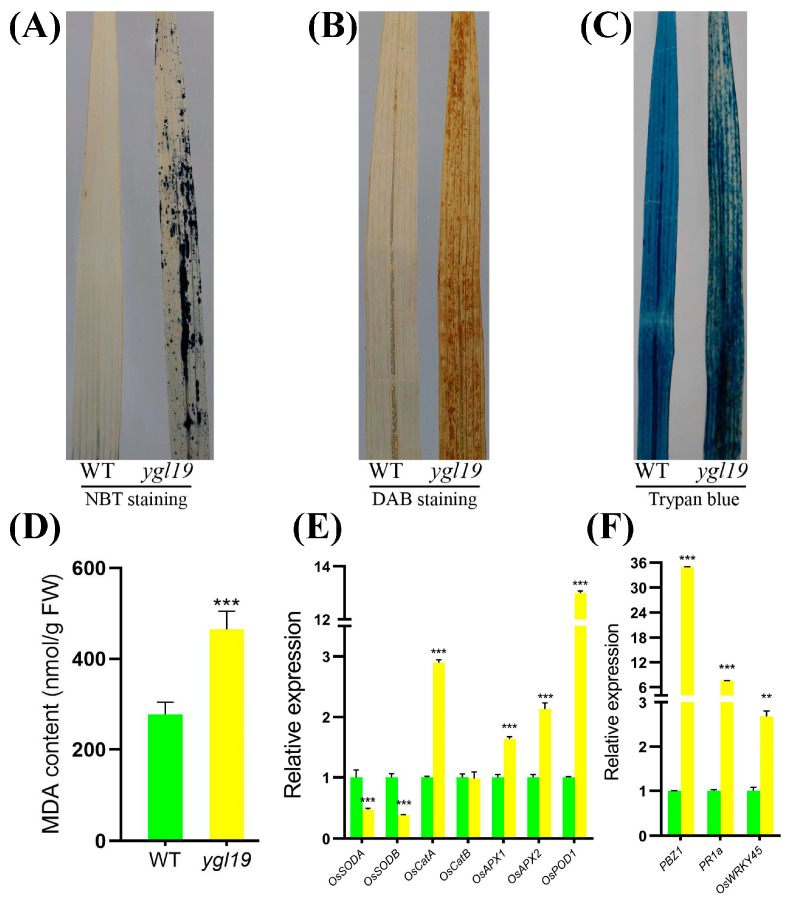
Phenotype of ROS accumulation in wild-type and *ygl19* mutant plant leaves at the tillering stage. (**A**–**C**) Histochemical detection of superoxide anions via NBT staining, hydrogen peroxide via DAB staining, and dead cells via trypan blue staining. (**D**) MDA content. (**E**) Relative expression of genes related to ROS scavenging. (**F**) Relative expression of ROS-responsive genes. WT, wild type. In (**D**–**F**), error bars represent the SDs of three biological replicates, and asterisks indicate statistically significant differences in the *ygl19* mutant compared with the wild type (** *p* < 0.005, *** *p* < 0.0005, Student’s *t*-test).

At the tillering stage, the *ygl19* mutant exhibited reddish-brown spots on the leaves. A previous study suggested that the formation of leaf spots is accompanied by a burst of reactive oxygen species (ROS) [32]. This prompted us to investigate whether ROS were accumulated in the *ygl19* mutant plant at the tillering stage. Nitro blue tetrazolium (NBT) staining was able to reflect the accumulation of superoxide anions via the formation of blue formazan precipitates. There were extensive leaf areas showing NBT staining in the *ygl19* mutant plant, whereas there was very little staining in the wild-type leaves (Figure 12A). 3,3′-diaminobenzidine (DAB) staining was used to indicate the accumulation of hydrogen peroxide. Intense brown staining by DAB was shown to correlate with spot formation in the *ygl19* mutant leaves, but such a signal was hardly seen in the wild-type leaves (Figure 12B). Trypan blue staining was performed to detect cell death, and it was found that the *ygl19* mutant had blue precipitates, while the wild type did not (Figure 12C). ROS accumulation would lead to lipid peroxidation and membrane damage and form MDA, an end product of oxidized lipids. At the tillering stage, the MDA content in the *ygl19* mutant leaves was also much higher than that in the wild type (Figure 12D). These results suggest that ROS like superoxide anions and hydrogen peroxide were accumulated in the *ygl19* leaves at the tillering stage, which further led to lipid peroxidation and leaf cell death.

Under oxidative stress, plants can activate the expression of antioxidative enzyme genes to remove ROS. SOD is a major enzyme for scavenging superoxide anions, while CAT, APX, and POD play important roles in eliminating hydrogen peroxide. The expression levels of these enzyme-encoding genes were additionally investigated in the *ygl19* mutant and wild-type leaves at the tillering stage via qRT-PCR. The results showed the expression of *OsSODA* and *OsSODB* to be downregulated in the *ygl19* mutant; meanwhile, *OsCatA*, *OsAPX1*, *OsAPX2,* and *OsPOD* were upregulated in the *ygl19* mutant (Figure 12E). These results suggest that genes encoding various ROS scavenging enzymes were regulated in the *ygl19* mutant, which might play important roles in regulating ROS metabolism in the mutant.

ROS bursts usually activate the responsive expression of defense signaling-related genes. Therefore, the expression of three defense signaling-related genes (*PBZ1*, *PR1a,* and *OsWRKY45*) was investigated in the *ygl19* and wild-type leaves at the tillering stage via qRT-PCR. The results showed that the expression levels of *PBZ1*, *PR1a*, and *OsWRKY45* were all upregulated in the *ygl19* leaves (Figure 12F). This molecular evidence implies that ROS in *ygl19* spot leaves could induce the responsive expression of defense signaling-related genes, which might lead to an enhanced defense response.

To verify whether the *YGL19* gene affects the defense response activated by ROS metabolism, the *ygl19* and wild-type plants were inoculated with the bacterial blight strain PXO99 at the tillering stage. However, the *ygl19* and wild-type plants showed no reaction to the inoculation of PXO99. After investigating, it was found that the wild-type “Shuangyinzhan” is a rice variety with first-level resistance to bacterial blight, which means it cannot be infected. Therefore, it cannot be judged whether the *YGL19* gene is related to the regulation of bacterial blight resistance.

## 3. Discussion

### 3.1. A Single-Base Substitution of the YGL19 Gene (LOC_Os03g21370) Is Responsible for Phenotype Variation in the ygl19 Mutant

The main mechanism of plant photosynthesis is in the leaves, so color variation in leaves will have an important impact on photosynthesis and even the growth and development of plants [33]. In recent years, leaf color mutants have been increasingly used in the study of plant functional genomes, the physiological and biochemical mechanisms of photosynthesis, and applications in genetics and breeding [34].

The analysis of leaf color mutation is an effective way to clarify the function of the genes involved in photosynthetic pigment metabolism. Here, a spontaneous yellow-green-leaf rice mutant, *ygl19*, showed a yellow-green phenotype throughout the entire growth period (Figure 1A,D), accompanied by deficient chlorophyll contents (Figure 1B,E). Moreover, there was a burst of reactive oxygen species at the tillering stage of the *ygl19* mutant (Figure 1H,I). These results suggest that the *YGL19* gene may be related to the regulation of chlorophyll synthesis. Leaf color mutants could change the physiological function of rice photosynthesis, which affects grain yield [35,36]. In this study, three agronomic traits, plant height, tiller number, and the total number of grains per plant, were affected in the *ygl19* mutant (Figure 2). Similar phenomena were found in the *ygl6* [37] and *ys83* [38] mutants in rice, but these were different from *ygl22,* which had unchanged agronomic characteristics (plant height, number of panicles per plant, and grain yield per plant) [39]. Through map-based cloning and complementation experiments, it was confirmed that the mutant phenotype of *ygl19* was induced as a consequence of a 1 bp substitution of the LOC_Os03g21370 gene (Figure 3, Figure 4 and Figure 5). To date, numerous studies have reported that yellow leaf color genes are localized in the chloroplasts, such as *OsIspF* [40], *YLWS* [41], and *WAL3* [33]. In the present study, *YGL19* had higher expression in the leaves and other green tissues (Figure 9A), and the YGL19 protein was localized in the chloroplasts (Figure 9B). Therefore, the rice leaf color gene *YGL19* was cloned in this study and might play an important role in chlorophyll metabolism, photosynthesis, and ROS metabolism based on the mutant phenotype of *ygl19*.

### 3.2. YGL19 Protein Homologues Are Unique for Photoautotrophs and Contain a Conserved Ycf54 Domain

Searching for and obtaining YGL19 homologs from 139 species retrieved from the NCBI website showed that these species were photoautotrophs. The evolutionary results showed that the YGL19 protein evolved from lower prokaryotic photoautotrophic bacteria and exhibited the closest evolutionary relationships with *Brachypodium distachyon*, *Zea mays*, *Hordeum vulgare,* and *Triticum aestivum* (Figure 6). Interestingly, these homologous sequences contain a conserved functional domain, “Ycf54”, which is the main component of YGL19 homologues in bacteria and protists and also the most important component in plants (Figure 7). The fully conserved amino acid proline (P at position 59) among all species was exactly in the middle of the Ycf54 domain (Figure 8).

A previous study indicated that in *Synechocystis* sp. PCC 6803, an auxiliary protein with Ycf54 and a catalytic subunit (AcsF/CycI) form an oxygen-dependent cyclase, which catalyzes the magnesium protoporphyrin IX monomethyl ester to the green product protochlorophyllide *a* [42]. In addition, it was recently proposed that extremely high concentrations of the cyclase substrate magnesium protoporphyrin IX monomethyl ester were accumulated in the *ycf54* gene deletion mutant of the cyanobacterium *Synechocystis* 6803 [22,43]. Similarly, Hollingshead et al. [44] confirmed that the *Synechocystis* 6803 *ycf54* mutant had decreased Mg-protoporphyrin IX methyltransferase and protochlorophyllide reductase activity, which indicated the role of the Ycf54 auxiliary factor. A further study with large-scale bioinformatic analysis indicated that Ycf54 played a role in the evolution of O_2_-dependent cyclase in *Prochlorococcus* [42]. Moreover, after in vivo heterologous activity analysis in the anoxygenic photosynthetic bacterium *Rubrivivax gelatinosus* and in the nonphotosynthetic bacterium *Escherichia coli*, Ycf54 proved to be an absolute requirement for oxygenic phototrophs [45]. It is speculated that this Ycf54 subunit had always been preserved during the course of evolution [46]. These studies support the conclusion that Ycf54 is a requisite component in the oxygen-dependent cyclase of photoautotrophs. To analyze the allelic variation in the *YGL19* gene in rice germplasm accessions, we searched the sequence variations in the coding region of the *YGL19* gene in 4726 rice accessions including *Indica*, *Japonica*, intermediate, and *Aus* [34,47,48], but only found three synonymous mutations in the exons of these rice variations, implying protein variation in the *YGL19* gene is not permitted in the process of rice domestication and breeding. Collectively, the above results indicate that the YGL19 protein homologs are conserved in eukaryotes and prokaryotes and are unique to photosynthetic organisms.

### 3.3. The Mutation of the YGL19 Gene Affects the Expression of Tetrapyrrole Biosynthesis-Related Genes, Thereby Regulating the Chlorophyll Content

It is recognized that tetrapyrroles play a key role in various biological processes, including photosynthesis and respiration. There are four different types of tetrapyrroles produced by higher plants: chlorophyll, heme, phytochromobilin, and siroheme [49]. Chlorophyll is a group of modified tetrapyrrole molecules that are characterized by their fifth isocyclic or E-ring, the C17 esterified geranyl/phytol part, and the magnesium ion chelated in the center [50]. Heme is a cyclic tetrapyrrole containing Fe^2 +^ and is a prosthetic group of many photosynthetic and respiratory proteins [51]. Tetrapyrrole biosynthesis includes the chlorophyll pathway and heme pathway. In the chlorophyll pathway, chlorophyll synthesis from L-glutamate to chlorophyll *b* requires a 17-step enzymatic reaction, and all 31 genes encoding these 17 enzymes have been identified in higher plants represented by *Arabidopsis* (*Arabidopsis thaliana*) [52,53]. In the heme pathway, heme synthesis from protoporphyrin-IX to phytochromobilin requires a three-step enzymatic reaction, and all seven genes encoding these three enzymes have also been identified in higher plants [52]. There are 10 genes from L-glutamate to protoporphyrin-IX: *OsGluRS*, *HEMA*1, *GSA*, *HEMB*, *HEMC*, *HEMD*, *HEME1*, *HEME2*, *HEMF,* and *HEMG*. And the branch from protoporphyrin-IX to chlorophyll *b* has 11 genes: *ChlD*, *ChlH*, *ChlI*, *GUN4*, *ChlM*, *CRD1*, *PORA*, *DVR1*, *YGL1*, *CAO1,* and *CAO2*.

*OsGluRS* encodes glutamyl-tRNA synthetase for the common steps of the tetrapyrrole biosynthetic pathway, transforming L-glutamate to glutamyl-tRNA [23]. *HEMA*1 encodes glutamyl-tRNA reductase, which is the first step of the tetrapyrrole biosynthetic pathway [54]. *GSA* encodes glutamate 1-semialdehyde and acts as the second step of the tetrapyrrole biosynthetic pathway [55]. And the rest of the seven genes that encode the enzymes for protoporphyrin IX are porphobilinogen synthase (*HEMB*), hydroxymethylbilane synthase (*HEMC*), uroporphyrinogen III synthase (*HEMD*), uroporphyrinogen decarboxylase (*HEME1* and *HEME2*), coproporphyrinogen oxidative decarboxylase (*HEMF*), and protoporphyrinogen oxidase (*HEMG*) [52]. Chelatase D subunit (*CHLD*), chelatase H subunit (*CHLH*), and magnesium chelatase I subunit (*CHLI*) are three subunits of magnesium chelatase that transform protoporphyrin IX to Mg-protoporphyrin IX [24]. *ChlM* encodes magnesium protoporphyrin IX methyltransferase, transforming Mg-protoporphyrin IX to Mg-protoporphyrin IX monomethyl ester [56]. And the rest of the six genes encoding the enzymes for chlorophyll *b* are magnesium-protoporphyrin IX monomethyl ester cyclase (*CRD1*) [57], protochlorophyllide oxidoreductase A (*PORA*) [2], 3,8-divinyl protochlorophyllide *a* 8-vinyl reductase (*DVR*) [25], chlorophyll synthase (*YGL1*), and chlorophyllide *a* oxygenase (*CAO1* and *CAO2*) [26,58]. We initially examined the expression levels of 21 genes associated with chlorophyll biosynthesis in rice, and most genes were downregulated in the *ygl19* mutant; only *HEMA*1, *HEMB*, *CAO1,* and *CAO2* were upregulated (Figure 10A). This might be the main factor that led to the decreased chlorophyll level in the *ygl19* mutant (Figure 1). In the heme pathway, *FCI* and *FCII* encode ferrochelatase 1 and 2, transforming protoporphyrin-IX to heme; *HO1* and *HO2* encode heme oxygenase 1 and 2, transforming heme to biliverdin IXa; and *HY2* encodes phytochromobilin synthase, transforming biliverdin IXa to phytochromobilin [52]. Four tetrapyrrole genes in the heme branch, FCII, HO1, HO2, and HY2 were decreased in the *ygl19* mutant; only *FCI* was upregulated (Figure 10B). It has been reported that an auxiliary protein, Ycf54, and a catalytic subunit (AcsF/CycI) form an oxygen-dependent cyclase, which catalyzes the magnesium protoporphyrin IX monomethyl ester to form protochlorophyllide *a* [42]. We speculated that the expression changes in tetrapyrrole biosynthesis-related genes might be caused by the lack of enzyme activity in the oxygen-dependent cyclase in the *ygl19* mutant, which could accumulate magnesium protoporphyrin IX monomethyl ester and cause subsequent regulation via the plastid–nucleus retrograde signaling pathway. These results indicated that *YGL19* can regulate the synthesis of photosynthetic pigments by regulating the expression of the tetrapyrrole synthesis pathway genes.

### 3.4. The Mutation of the YGL19 Gene Affects the Expression of Photosynthesis-Related Genes, Thereby Regulating Photosynthetic Capacity

Photosynthesis plays a vital role in plant growth and morphogenesis [59]. In our study, the net photosynthesis rate was significantly lower in the *ygl19* mutant than that in the wild type (Figure 1G). Photosynthesis-related genes are usually divided into nucleus- and plastid-encoded genes. This study analyzed the expression of seven nucleus-encoded genes encoding the chlorophyll *a*/*b* binding protein (*Cab1R* and *Cab2R*), light-harvesting chlorophyll *a*/*b*-binding protein of PSII (*LHca* and *LHcb*), PSII reaction center protein (*PSB28*), Rubisco small subunit (*RBCS*), and nuclear-encoded RNA polymerase (*RpoTp*). *Cab1R*, *Cab2R*, *LHca,* and *LHcb* are chlorophyll a/b binding protein genes, which are affected by light pulses [60]. Studies have shown that the light-harvesting protein *LHca* can participate in light-harvesting activities as well as chloroplast metabolism [61]. PSB28 is a soluble protein in the PSII complex, and the *Psb28* gene positively regulates drought tolerance in wheat [62]. In photosynthesis and photorespiration, Rubisco catalyzes the first step of CO_2_ assimilation, and it is a decahexameric protein consisting of eight small subunits (RBCS) and eight large subunits (RBCL) in higher plants [63]. Nuclear-encoded RNA polymerase is a single-subunit bacteriophage-type enzyme, which mainly transcribes plastid genes for the transcription/translation apparatus. It has been reported that nuclear-encoded RNA polymerase is activated at a strictly limited stage of leaf development and controlled by processes following transcription, including mRNA stabilization, translation, and translocation [64]. In this study, three nucleus-encoded genes (*Cab2R*, *LHca,* and *RpoTp*) were downregulated, while two genes (*Cab1R* and *RBCS*) were upregulated in the *ygl19* mutant (Figure 11A).

Moreover, 14 plastid-encoded genes were detected, encoding the subunit of PSI (*psaA*, *psaB,* and *psaC*), core component of PSII (*psbA*, *psbB,* and *psbC*), NADH dehydrogenase subunit (*ndhA* and *ndhB*), cytochrome *b_6_f* complex subunit (*petD*), ATP synthase complex subunit (*atpA* and *atpB*), Rubisco large subunit (*rbcL*), and plastid-encoded RNA polymerase subunit (*rpoA* and *rpoB*). PSI is located primarily on the monolayer stromal thylakoid membrane, while PSII is mainly located on the stack of grana of the thylakoid membrane, both constituting important sites of bioluminescence [65]. The genes of PSI and PSII (*psaA*, *psaB*, *psaC*, *psbA*, *psbB,* and *psbC*) exhibited different degrees of lower gene expression in the *ygl19* mutant (Figure 11B). Our result is consistent with the findings of the *Arabidopsis thaliana high chlorophyll fluorescence145* (*hcf145*) mutation, which leads to the reduced stability of the plastid tricistronic psaA-psaB-rps14 mRNA and PSI deficiency level [66]. NADH dehydrogenase has type I (NDH-1; corresponds to *ndhA*) and II (NDH-2; corresponds to *ndhB*), which are multi-subunit complexes similar to mitochondrial complex I and single-subunit flavoenzyme, respectively. They can reduce the plastoquinone in a nonphotochemical manner, which is an important part of electron transport chains between PSI and PSII [67]. It has been reported that *petD* can directly bind to the *psbB* operon (*psbB*-*psbT*-*psbH-petB*), which is a typical polycistronic transcription unit in chloroplasts. The *petD* gene encodes subunit IV of the cytochrome *b_6_f* complex, one of the thylakoid membrane complexes [68,69]. Chloroplast ATP synthase is a crucial protein complex associated with the thylakoid membrane that plays a role in the light-dependent reactions of photosynthesis. ATP synthase major subcomplex CF_1_ is a membrane-extrinsic, soluble subcomplex, which consists of five subunits (α, β, γ, δ, and ε). And the α and β subunits are encoded by the plastid genes *atpA* and *atpB*, respectively [31,70]. In the chloroplast, the large subunit is encoded by *RBCL*, and it catalyzes two competing reactions, CO_2_ fixation in photosynthesis and the production of 2-phosphoglycolate in the photorespiratory pathway [71]. In rice and tobacco, deletion of the *rpoA* or *rpoB* encoding the subunits of plastid-encoded RNA polymerase resulted in photosynthetic incompetence [33,72]. In our study, plastid-encoded photosynthesis-related genes showed lower expression levels in the *ygl19* mutant. Liang et al. [73] found that five plastid-encoded genes (*psaA*, *psaB*, *petA*, *atpA,* and *rpoB*) were fewer in a *csl1* mutant, which exhibited a chlorotic seedling phenotype before the trefoil stage. And they also reported that seven genes (*psaA*, *psaB*, *psbA*, *psbB*, *petD*, *atpA,* and *rbcL*) were significantly decreased in the *ygl8* mutant with a yellow-green leaf phenotype [28]. We speculated that the downregulated expression of these photosynthesis-related genes might be caused by the monitored regulation of chlorophyll deficiency or chloroplast function through the plastid–nucleus retrograde signaling pathway. These results imply that the *YGL19* gene can regulate leaf photosynthesis by regulating the expression of nucleus- and plastid-encoded photosynthesis-related genes.

### 3.5. The Mutation of the YGL19 Gene Affects the Expression of ROS Pathway-Related Genes, Thereby Regulating the Metabolism of ROS

Alterations in tetrapyrrole homeostasis might cause a redox imbalance in photosynthetic cells, and ROS would generate in this condition [13]. ROS-mediated redox signaling plays an important role in regulating rice growth [74]. The accumulation of ROS can also damage cells and negatively affect organisms, and oxidative stress signaling can be induced by ROS molecules in cells to protect or mitigate damage [75]. ROS comprise many species, such as singlet oxygen (^1^O_2_), superoxide anions (O_2_^−^), hydroxyl radicals (OH) and hydrogen peroxide (H_2_O_2_), which affect proteins, lipids, and DNA repairment, and they simultaneously impact gene expression as signals [76]. The pattern of nitro blue tetrazolium (NBT) staining reflected the formation of blue formazan precipitates and was indicative of O_2_^−^ accumulation in the leaves of the *ygl19* mutant compared to the wild type (Figure 12A). The 3,3′-diaminobenzidine (DAB) staining reflected the brown precipitates and was indicative of H_2_O_2_ accumulation in the leaves of the *ygl19* mutant (Figure 12B). MDA is the product of membrane lipid peroxidation, and a change in its content can be used as an indicator to measure the degree of oxidative damage to plants, indirectly reflecting the antioxidant capacity of plant tissues [49]. The MDA content was greatly increased in the *ygl19* mutant compared to the wild type (Figure 12D). The accumulation of MDA causes cellular membrane damage and indirectly affects cell death [77]. Here, we also found that the *ygl19* mutant had a mosaic pattern of positive staining of trypan blue, indicative of dead cells (Figure 12C). O_2_^−^ is produced in the chloroplasts and is unstable. Superoxide dismutase catalyzes O_2_^−^ into H_2_O_2_, mainly encoded by *OsSODA* and *OsSODB*. Next, H_2_O_2_ is hydrolyzed by peroxidases (encoded by *OsAPX* and *OsPOD*) or catalases (encoded by *OsCAT*) to produce H_2_O and O_2_ [78]. Through qRT-PCR, we found that the expression of the antioxidative enzyme genes *OsCATA*, *OsAPX1*, *OsAPX2,* and *OsPOD1* significantly increased in the *ygl19* mutant, while *OsSODA* and *OsSODB* decreased (Figure 12E). ROS probably also play a key role in defense responses, accompanied by the induction of defense response-related genes [78]. Therefore, we detected three ROS-responsive defense response-related genes (*PBZ1*, *PR1a,* and *OsWRKY45*), which were all upregulated in the *ygl19* mutant (Figure 12F). It is speculated that ROS dramatically increased in the *ygl19* mutant due to alterations in tetrapyrrole homeostasis and/or the fact that the photosynthetically active reaction center was overexcited as a result of a functional defect in the chloroplasts. ROS act as second messengers involved in the transmission of oxidative stress signaling, and this may be the reason for the expression changes in ROS metabolism-related genes. These results imply that the *YGL19* gene can regulate the content of ROS in leaves by regulating the expression of genes related to ROS metabolism.

## 4. Materials and Methods

### 4.1. Plant Materials and Growth Conditions

The *ygl19* mutant was derived from the rice cultivar “Shuangyinzhan” (*Oryza sativa* L. subsp. *indica*), and its mutant characters were inherited stably after multiple generations of self-breeding. All materials used in this study were grown in paddy fields under natural conditions in Nanchang, Jiangxi Province, China. The phenotype of plants was visually observed during the whole developing period.

### 4.2. Measurement of Photosynthetic Pigments

Leaf samples were collected from the *ygl19* mutant and its wild type at the seedling stage and tillering stage. After grinding into powder with liquid nitrogen, 0.2 g of leaf powder was used to extract pigments with 80% acetone at 4 °C for 2.5 h under dark conditions. Then, the contents of chlorophyll (Chl) and carotenoids (Caro) were measured using an INFINITE 200 PRO (Tecan, Männedorf, Switzerland) at 470 nm, 646 nm, and 663 nm according to the method as described previously [79].

### 4.3. Measurement of the Net Photosynthetic Rate

The net photosynthetic rate (Pn) was measured under conditions of 400 ppm CO_2_ concentration and sunny weather, between 09:30 and 10:30 a.m. when the solar radiation was approximately 1200 μmol m^−2^ s^−1^. At the late tillering stage, the penultimate fully expanded leaves of the wild type and *ygl19* mutant were selected to measure the photosynthetic capability with a portable photosynthetic apparatus Li-6400 (LI-COR, Hainesport, NJ, USA). Three biological replicates were performed for the wild type, *ygl19* mutant, and transgenic plants.

### 4.4. Map-Based Cloning of the YGL19 Gene

For genetic population construction, the *ygl19* mutant was crossed with the rice cultivars 02428 (*Oryza sativa* L. subsp. *japonica*) and 9311 (*Oryza sativa* L. subsp. *indica*). The resulting F_1_ grains were sown and self-crossed to construct the F_2_ populations, which were used for genetic analysis and gene mapping.

A total of 44 F_2_ mutant plants from the cross-population of the *ygl19* mutant and 9311 were randomly selected to preliminarily map the *YGL19* gene using simple sequence repeat markers well distributed on all 12 chromosomes and distinguishing two parents. For fine mapping, nine additional In/Del primers were successfully designed to distinguish the *ygl19* mutant and 02428 based on the published genomic sequence differences between the *indica* and *japonica* rice varieties. Therefore, the fine mapping was performed by using these nine In/Del primers and 1062 F_2_ mutant individuals from the cross-population of the *ygl19* mutant and 02428. The primer sequences used are listed in Supplementary Table S1.

### 4.5. Complementation of the YGL19 Gene

For the complementation of the *YGL19* gene into the *ygl19* mutant, the 621 bp coding sequence of the wild-type *YGL19* gene (LOC_Os03g21370) was cloned into the binary vector pBWA(V)HU, using primers 5′-CAGTGGTCTCACAACATGGTGGCTCCCGCGACGCT-3′ and 5′-CAGTGGTCTCATACACTATGCTGTTCCATTGCTAG-3′ with the *Bsa*I restriction site. The fusion vector pBWA(V)HU-Ubi-*YGL19* driven by the ubiquitin promoter was transformed into *ygl19* calli via *Agrobacterium*-mediated transformation, as described previously [80]. Briefly, mature *ygl19* seeds were used for the induction of calli. Then, the calli were sub-cultured and inoculated with *Agrobacterium tumefaciens* EHA105 containing pBWA(V)HU-Ubi-*YGL19* vector. A screening medium with hygromycin resistance was used to screen the successfully transfected positive callus tissues, which were used to induce seedling formation through dedifferentiation culture and root formation through rooting culture. The T_0_ transgenic rice plants were grown in the field and the seeds were harvested. The positive transgenic T_1_ seeds were screened out via hygromycin treatment. Transgenic plants were also detected using *Hyg* primers F: 5′-CTGCCCGCTGTTCTACAACCGG-3′ and R: 5′-GGAGCATATACGCCCGGAGTC-3′, which specifically amplify the screening resistance gene hygromycin.

To examine the single-base alternation in the *ygl19* mutant, wild type, and transgenic lines, a dCAPs molecular marker primer (F: TTCATGCTGGACGAGGAG, R: TTGGGGAACCTGTCGAGGAACTTCG) was designed to amplify a 128 bp fragment with or without the *Taq* I restriction site based on the SNP alternation of *ygl19*, by using the dCAPS Finder 2.0 Program (http://helix.wustl.edu/dcaps/dcaps.html, accessed on 6 September 2022). The *Taq* I enzyme could match the sequence (T^▼^CGA) in the *ygl19* amplicon; then, the amplified 128 bp fragment could be digested into 105 bp and 23 bp fragments. The wild-type amplicon product without this enzyme digestion site could not be digested, showing a 128 bp fragment. The T_0_ transgenic complementary lines have both *ygl19* and *YGL19* sequences, and we could detect 128 bp and 105 bp fragments.

### 4.6. Gene Expression Analysis via qRT-PCR

Total RNA was extracted with a TRIZOL Kit (Invitrogen, Carlsbad, CA, USA) according to the instructions. A total of 1.0 mg of RNA was used for cDNA synthesis using the PrimeScript RT kit and gDNA Eraser (Takara, Dalian, China) according to the manufacturer’s protocol. Primers were designed using Primer3 online tools (https://bioinfo.ut.ee/primer3-0.4.0/, accessed on 14 September 2021) and synthesized by Beijing Tsingke Biotech Co., Ltd. (Tsingke, Beijing, China). Real-time quantitative PCR (qRT-PCR) was performed in a total volume of 10 μL containing 0.2 μM of each primer pair and 1 × SYBR green PCR master mix (Tiangen Biotech, Beijing, China) by using the CFX96 real-time PCR system (Bio-Rad, Hercules, CA, USA). All reaction conditions were as follows: 95 °C for 15 min; 40 cycles of 95 °C for 10 s, 60 °C for 10 s, and 72 °C for 15 s; and then a melt curve from 65 to 95 °C. For each experimental group, qRT-PCR was operated with three technical replicates for each of three biological replicates. The 2^−ΔΔCT^ method was applied to calculate the quantitative expression of each gene relative to the internal control. The rice genes *ARF* (LOC_Os05g41060), *Profilin-2* (LOC_Os06g05880), and *EF-1α* (LOC_Os03g08020) were used as the reference genes as previously described [81]. All qRT-PCR primers are listed in Supplementary Table S2.

### 4.7. Subcellular Localization

The full-length cDNA fragment of the *YGL19* gene was amplified from the wild type and inserted into the pBWA(V)HS-GLosgfp vector. The PCR primers were 5′-CAGTGGTCTCACAACATGGTGGCTCCCGCGACGCT-3′ and 5′-CAGTGGTCTCATACACTATGCTGTTCCATTGCTAG-3′, which separately contained a *Bsa*I site at the 5′-end and a *EcoR*I site at the 3′-end of the cDNA fragment. The constructed fusion vector pBWA(V)HS-*YGL19*-GLosgfp and the empty vector pBWA(V)HS-GLosgfp (negative control) were separately transformed into the rice protoplasts, following a previously described method [28]. Finally, the GFP fluorescence in the transformed protoplasts was examined under a laser scanning confocal microscope (Fluoview FV1000, Olympus, Tokyo, Japan).

### 4.8. Determination of Reactive Oxygen Species (ROS)

The same parts of the fully expanded leaves of the *ygl19* mutant with leaf spots and wild type at the tillering stage were selected for experiments. We put the leaves in a 15 mL tube containing NBT staining solution (0.5 ng/mL in 0.01 M PBS (PH = 7.6)) and vacuumized. The leaves were immersed in the bottom of the tube and stained for more than 3 h at 28 °C in the dark. The leaves were taken out and placed in a centrifuge tube containing 80% ethanol, boiled in a water bath until the green pigments were removed, and finally stored in 70% glycerol at 4 °C and photographed [82,83,84].

Hydrogen peroxide (H_2_O_2_) in the rice leaves was detected using 3,3′-diaminobenzidine (DAB) staining as previously described [85]. Leaves were placed in a 15 mL tube containing DAB staining solution (1 mg/mL) and vacuumized. The leaves were taken out and placed in a centrifuge tube containing 80% ethanol, boiled in a water bath until the green pigments were removed, and finally stored in 70% glycerol at 4 °C and photographed [82,86].

For trypan blue staining solution, the leaves of wild type and *ygl19* mutant were soaked in trypan blue staining solution (LPTB; 2.5 mg mL^−1^ trypan blue, 25% (W/V) lactic acid, 23% water-saturated phenol, and 25% glycerol in H_2_O), stained in a boiling water bath for 2 min, stained overnight at room temperature after natural cooling, dissolved in 2.5 g/mL chloral hydrate for 3 days to decolorize (with the decolorizing solution changed once a day), and finally stored in 70% glycerol at 4 °C [87].

The activities of malondialdehyde (MDA) in the extracts were measured using the following kits from Beyotime Biotechnology (Beyotime Biotechnology, Beijing, China) according to the manufacturer’s instructions: The extracts of leaves were prepared using the cell lysis buffer for Western blotting and IP (Cat. P0013, Beyotime Biotechnology, Beijing, China). The concentrations of proteins were detected using the Enhanced BCA Protein Assay Kit (Cat. P0012S, Beyotime Biotechnology, Beijing, China). The content of malondialdehyde (MDA) was detected using the Lipid Peroxidation MDA Assay KIT (Cat. S0131, Beyotime Biotechnology, Beijing, China) [88].

### 4.9. Data Sources

The protein sequences, transcript sequences, genomic sequences, and GFF annotation files of 106 green plants, 16 protists, and 17 bacteria were downloaded from the Phytozome (https://phytozome-next.jgi.doe.gov/, accessed on 16 November 2022) and NCBI (https://blast.ncbi.nlm.nih.gov/Blast.cgi, accessed on 16 November 2022) websites.

### 4.10. Phylogenetic Tree Construction and Multiple Sequence Alignment

The protein sequence of the YGL19 protein was input under Protein BLAST by logging onto the NCBI website, and its homologous proteins in different species were downloaded. The software Clustal X (v2.1) was used for amino acid sequence alignment. The neighbor-joining method in MEGA (v11.0.13) software was adopted to build an evolutionary tree. Bootstrapping was conducted 1000 times to build an evolutionary tree for homologous proteins. The tree was visualized using the Interactive Tree of Life (iTOL) and FigTree v1.4.4.

### 4.11. Analysis of Gene Structure and Sequence Motifs

The coding sequence (CDS) information of the 139 full-length primary protein sequences from green plants, chlorophytes, protists, and bacteria was retrieved from the GFF annotation files and submitted to TBtools v2.010 [89] to visualize the exon–intron organization and protein domain of YGL19 homologous proteins in representative species. Motif analysis was performed using MEME suite v5.3.0 [90], which scans for motifs recurring in a set of sequences. Motif analysis was carried out using the MEME server (https://meme-suite.org/meme/tools/meme, accessed on 12 September 2022), keeping the width of the motif at 6–50 amino acids, the number of motifs at 20, and the other parameters set to default. The gene structure and conserved motif patterns were visualized using TBtools v2.010 [89].

### 4.12. Statistical Analysis

All data were statistically analyzed using Microsoft Excel 2021 (Microsoft Corporation, Washington, DC, USA) and GraphPad Prism 5 (v5.01) (GraphPad Software, Inc., San Diego, CA, USA). Student’s *t*-test was conducted to compare the wild type and the *ygl19* mutant, and *, **, and *** represent significant differences at the 0.05, 0.005, and 0.0005 levels, respectively. GraphPad Prism 5 was used to construct graphs.

## 5. Conclusions

In conclusion, this study identified the *YGL19* gene (LOC_Os03g21370) in rice, responsible for the yellow-green leaf, spot leaf, and weakened agronomic characters of the *ygl19* mutant. The *YGL19* gene is expressed mainly in the leaf tissue and localizes in the chloroplasts to perform biological roles. YGL19 homologs are unique for photoautotrophs and contain a conserved YCF54 domain. The expression levels of tetrapyrrole synthesis-related genes, photosynthesis-related genes, and ROS metabolism-related genes were regulated in the *ygl19* mutant. It is suggested that the *YGL19* gene plays an important role in tetrapyrrole synthesis, photosynthesis, and ROS metabolism in rice.

## Figures and Tables

**Table 1:** Segregation of F_2_ populations from two crosses.

Cross	Wild-Type Green Leaf	Yellow-Green and Spotted Leaf	χ^2^ (3:1)	*p* Value
*ygl19*/9311	144	44	0.18	0.92
*ygl19*/02428	3173	1062	0.01	0.99

Note: Cross, female/male. The phenotype was determined via visual inspection. χ^2^ < χ^2^_0.05_ = 3.84 is for 3:1 segregation ratio. *p* > 0.05 is considered as significant.

**Table 2:** Natural variants of the *YGL19* gene in 4726 rice accessions.

SNP Position	Primary Allele	Secondary Allele	Primary Allele Frequency	Amino Acid Mutation and Position	snpEff Annotation
28	A	G	99.90%	no	5_prime_UTR_variant
180	T	G	60.40%	Ala16Ala	synonymous_variant
321	C	T	60.30%	Asp63Asp	synonymous_variant
559	A	G	59.90%	Val111Val	synonymous_variant
742	A	T	99.90%	no	intron_variant
764	A	C	93.60%	no	intron_variant
853	C	T	99.70%	no	intron_variant
1304	T	C	59.90%	no	intron_variant
1391	A	G	60.10%	no	intron_variant
1420	T	C	60.00%	no	intron_variant
1508	G	A	60.30%	no	intron_variant
1686	G	A	94.40%	no	intron_variant
1748	G	A	99.80%	no	intron_variant
1817	C	T	99.90%	no	intron_variant
1918	C	T	99.80%	no	intron_variant
2000	T	C	60.00%	no	intron_variant
2226	T	C	59.90%	no	intron_variant
2243	A	T	91.30%	no	intron_variant
2351	G	A	99.90%	no	intron_variant
2427	G	A	98.90%	no	intron_variant
2492	G	A	99.90%	no	intron_variant
2520	G	A	99.30%	no	intron_variant
2732	T	C	59.90%	no	intron_variant
2917	T	C	99.20%	no	splice_region_variant&intron_variant
3212	T	G	60.20%	no	3_prime_UTR_variant
3405	C	A	96.40%	no	3_prime_UTR_variant

## Data Availability

Data are contained within the article and Supplementary Material.

## Supplementary Materials

The following supporting information can be downloaded at: https://www.mdpi.com/article/10.3390/ijms242316762/s1.
