# Supplementary figures and images for: Yellow-Green Leaf 19 Encoding a Specific and Conservative Protein for Photosynthetic Organisms Affects Tetrapyrrole Biosynthesis, Photosynthesis, and Reactive Oxygen Species Metabolism in Rice
Source: Int J Mol Sci. 2023 Nov 25;24(23):16762. doi: 10.3390/ijms242316762 (PMC10706213; doi:10.3390/ijms242316762)

1 2 3 4 5 6 7 8 9 10 + M - O

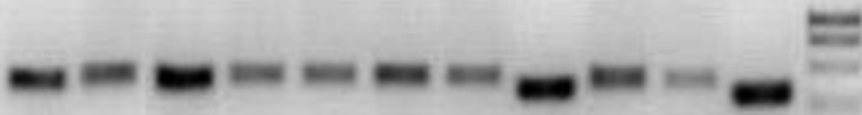

Supplement: Supplementary file 1 [file ijms-24-16762-s001.zip › Supplementary Figure S1.pdf]
